# Social Determinants of Neurodevelopmental Disorders: Associations with ADHD and ASD Among U.S. Children

**DOI:** 10.3390/children13010062

**Published:** 2025-12-31

**Authors:** Chinedu Izuchi, Chika N. Onwuameze, Godwin Akuta

**Affiliations:** 1 Avera Hospitals, 132 N Dakota Ave, Sioux Falls, SD 57104, USA; 2 Department of Developmental and Higher Education Studies, Grambling State University, Grambling, LA 71245, USA; 3 Texas Health and Human Services Commission, Lubbock, TX 79424, USA

**Keywords:** attention-deficit/hyperactivity disorder (ADHD), autism spectrum disorder (ASD), comorbidity, social determinants of health, socioeconomic gradients, neighborhood context, diagnostic disparities, health equity, National Survey of Children’s Health

## Abstract

**Background:** Attention-deficit/hyperactivity disorder (ADHD) and autism spectrum disorder (ASD) are prevalent neurodevelopmental conditions in childhood. Beyond biological factors, social and environmental conditions influence developmental experiences and pathways to diagnosis. Nationally representative studies examining multiple social determinants in relation to ADHD, ASD, and comorbidity across recent years remain limited. **Methods:** We analyzed pooled cross-sectional data from six cycles (2018–2023) of the U.S. National Survey of Children’s Health, including 205,480 children aged 3–17 years. Parent-reported, clinician-diagnosed current ADHD and ASD were the primary outcomes; comorbid ADHD and ASD were examined secondarily. Social determinants included household income relative to the federal poverty level, parental education, health insurance type, food insecurity, and caregiver-reported neighborhood safety. Survey-weighted prevalence estimates and logistic regression models accounted for the complex sampling design and adjusted for demographic, family, regional, and temporal factors. Results: The weighted prevalence of ADHD was 9.7% and ASD was 2.9%; 1.1% of children had comorbid ADHD and ASD. Lower household income, food insecurity, unsafe neighborhood conditions, and lower parental education were associated with higher adjusted odds of both conditions. Boys had substantially higher odds of ADHD and ASD. After adjustment, non-Hispanic Black and Hispanic children had lower odds of ASD than non-Hispanic White children, consistent with differential identification rather than lower underlying prevalence. Comorbidity was concentrated among socially disadvantaged children. **Conclusions:** ADHD and ASD are socially patterned across U.S. children. Integrating developmental screening with assessment of social risks may support more equitable identification and intervention.

## 1. Introduction

Neurodevelopmental disorders represent a significant public health concern in childhood, with long-term implications for cognitive functioning, educational attainment, mental health, and social participation across the course of life. Among these conditions, attention-deficit/hyperactivity disorder (ADHD) and autism spectrum disorder (ASD) are the most prevalent and widely studied due to their early onset, persistence into adulthood, and substantial functional impact on children and families [1,2,3]. Recent national estimates indicate that approximately one in ten U.S. children has been diagnosed with ADHD. At the same time, the prevalence of ASD has increased to nearly three percent, reflecting both improved detection and ongoing developmental burden [4,5].

Although ADHD and ASD are defined by neurobehavioral characteristics with strong genetic and neurobiological underpinnings, there is increasing recognition that these conditions are embedded within broader social and structural contexts. The social determinants of health framework emphasizes that children’s developmental trajectories are shaped not only by biology but also by the social, economic, and environmental conditions in which they are born and raised [6]. Family socioeconomic position, access to health care, neighborhood environments, and material stability can influence exposure to stress, opportunities for enrichment, and pathways to identification and support. These factors may shape both the manifestation of developmental difficulties and the likelihood that concerns are recognized, evaluated, and formally diagnosed within clinical or educational systems.

Socioeconomic disadvantage has been consistently associated with adverse developmental and behavioral outcomes in children. Chronic exposure to poverty-related stressors, including financial instability, food insecurity, and constrained access to supportive resources, is linked to difficulties in attention regulation, emotional control, and adaptive functioning [7,8,9]. Prior studies have shown that children from lower-income households or families with lower parental education experience higher ADHD symptom burden, greater functional impairment, and disparities in continuity of care [10,11]. Evidence regarding ASD has been more heterogeneous, with several studies reporting higher diagnosis rates among socioeconomically advantaged families, a pattern widely interpreted as reflecting differential access to diagnostic services rather than actual differences in underlying prevalence [12,13].

Neighborhood context represents an additional dimension through which social conditions may shape child development. Exposure to unsafe or disordered neighborhoods can limit opportunities for outdoor play, peer interaction, and independent exploration, while increasing caregiver stress and vigilance [14]. Caregiver perceptions of neighborhood safety, although subjective, capture lived environmental conditions that may influence children’s behavior and parental concern and help-seeking. Material hardship, including household food insecurity, has similarly been associated with poorer developmental and behavioral outcomes, reflecting both nutritional risk and broader household instability [7,15].

Patterns of diagnosis and service use for ADHD and ASD are also influenced by racial and ethnic inequities within health and educational systems. Numerous studies have documented that non-Hispanic Black and Hispanic children are diagnosed with ASD later or less frequently than non-Hispanic White children, even after accounting for socioeconomic factors [16,17,18]. These disparities are widely attributed to differences in access to developmental screening, specialty care, school-based evaluations, and culturally responsive services rather than differences in underlying neurodevelopmental risk [12,19]. For ADHD, racial and ethnic patterns are more complex, shaped by variation in school expectations, behavioral norms, referral practices, and structural bias within diagnostic systems.

Comorbidity between ADHD and ASD further complicates the clinical and public health landscape. Co-occurring ADHD and ASD are associated with greater functional impairment, higher service needs, and more complex care trajectories than either condition alone [3]. Despite its importance, comorbidity is often underexamined in population-based studies, particularly in relation to social determinants of health. Understanding whether children with comorbid conditions are disproportionately concentrated in socially disadvantaged contexts is essential for identifying groups with heightened developmental vulnerability and unmet support needs.

National surveillance systems such as the National Survey of Children’s Health (NSCH), the National Health Interview Survey, and the Autism and Developmental Disabilities Monitoring Network provide essential data for examining the population distribution of ADHD and ASD in the United States [4,5,20]. However, much of the existing literature relies on single-year analyses or examines social determinants in isolation. Fewer studies have leveraged multiple consecutive NSCH cycles to simultaneously examine income, parental education, insurance coverage, food insecurity, and neighborhood conditions in relation to ADHD, ASD, and their comorbidity while fully accounting for the survey’s complex sampling design [21,22].

To address these gaps, the present study uses pooled data from six consecutive NSCH cycles (2018–2023) to examine associations between key social determinants of health and ADHD and ASD among U.S. children aged 3–17 years. By combining multiple survey years, this analysis improves statistical precision and allows assessment of consistent social gradients across demographic and socioeconomic strata. Rather than implying causation, the study focuses on identifying population-level patterns that may inform equity-focused screening, service delivery, and policy efforts. The specific objectives were to: (1) estimate nationally representative prevalence of ADHD, ASD, and comorbid ADHD and ASD across social and demographic groups; (2) examine adjusted associations between selected social determinants and each condition; and (3) assess whether observed disparities persist after accounting for demographic, family, geographic, and temporal factors.

## 2. Materials and Methods

### 2.1. Study Design and Data Source

This study employed a cross-sectional, population-based design using pooled data from six consecutive cycles (2018–2023) of the U.S. National Survey of Children’s Health (NSCH). The NSCH is a nationally representative survey administered annually by the U.S. Census Bureau under the direction of the Health Resources and Services Administration (HRSA) to assess the physical, emotional, and developmental health of non-institutionalized children in the United States [1]. The survey uses an address-based sampling frame and randomly selects one child per sampled household for a detailed caregiver-reported assessment.

Pooling multiple NSCH cycles was undertaken to improve statistical precision, increase power for subgroup and comorbidity analyses, and allow examination of consistent social gradients across recent years. This approach is consistent with HRSA analytic guidance and prior peer-reviewed NSCH-based research [2,3].

### 2.2. Study Population

The analytic sample included children aged 3–17 years at the time of survey completion. Children younger than three years were excluded because ADHD and ASD diagnoses are less reliably identified in infancy and toddlerhood, and diagnostic criteria typically require sustained observation across developmental contexts [4].

Across the six pooled cycles, approximately 212,000 unweighted child records were available. Exclusion criteria included: (1) missing caregiver report of current ADHD or ASD status; (2) age outside the eligible range; (3) missing data on key social determinants of interest (household income, parental education, or neighborhood safety); and (4) missing survey design variables required for complex survey estimation. After exclusions, the final analytic sample comprised 205,480 children, representing approximately 73.1 million U.S. children nationally after application of survey weights.

### 2.3. Measures

#### 2.3.1. Outcomes

The primary outcomes were current attention-deficit/hyperactivity disorder (ADHD) and current autism spectrum disorder (ASD), based on caregiver report of clinician diagnosis. Caregivers were asked whether a health care provider had ever told them that the child had ADHD or ASD and whether the condition was current at the time of the survey. Binary indicators were created for current ADHD and current ASD, consistent with prior NSCH-based studies [5,6].

For secondary analyses, children were classified into mutually exclusive diagnostic categories: ADHD only, ASD only, or comorbid ADHD and ASD. Among children with ADHD or ASD, caregivers also rated condition severity as mild, moderate, or severe. These severity measures were used in sensitivity analyses to examine social gradients in caregiver-perceived impairment.

#### 2.3.2. Social Determinants of Health

Social determinants were selected a priori based on established conceptual frameworks and empirical literature on child development and health inequities [7].

Household income was categorized relative to the federal poverty level (FPL) using NSCH-provided income-to-poverty ratios (<100% FPL, 100–199% FPL, 200–399% FPL, and ≥400% FPL).

Parental education was defined as the highest educational attainment of any parent or guardian in the household and categorized as less than high school, high school diploma or equivalent, some college, or bachelor’s degree or higher.

Health insurance status at the time of survey completion was categorized as private insurance, public insurance (Medicaid or Children’s Health Insurance Program), or uninsured.

Household food insecurity was measured using caregiver responses indicating that the household sometimes or often could not afford enough food in the past 12 months. This measure has been widely used as an indicator of material hardship in population-based child health research [8].

Neighborhood safety was assessed using caregiver-reported perceptions of how safe the neighborhood is for children to play outside, categorized as “definitely safe,” “somewhat safe,” or “not safe.” Although subjective, this measure captures lived environmental conditions relevant to child development and caregiver stress [9].

### 2.4. Covariates

Covariates were selected based on prior literature and included child age (continuous), sex (male or female), race/ethnicity (non-Hispanic White, non-Hispanic Black, Hispanic, other or multiracial), family structure (two-parent household, single-parent household, or other arrangement), U.S. Census region (Northeast, Midwest, South, West), and survey year. Survey year was included to account for temporal variation, including potential changes in health care access and service delivery during the COVID-19 pandemic period [10].

### 2.5. Statistical Analysis

All analyses accounted for the NSCH’s complex sampling design. Survey-provided weights, strata, and primary sampling units were applied to produce nationally representative estimates and valid standard errors. Pooled survey weights were constructed in accordance with HRSA guidance to ensure appropriate weighting across combined survey years [2].

Weighted descriptive statistics were used to summarize demographic, socioeconomic, and neighborhood characteristics of the study population. Prevalence estimates for ADHD, ASD, and comorbid ADHD and ASD were calculated overall and across categories of social determinants. Group differences were assessed using Rao–Scott chi-square tests for categorical variables and survey-adjusted t-tests for continuous variables.

Associations between social determinants and ADHD or ASD were examined using survey-weighted logistic regression models. Separate multivariable models were fitted for ADHD and ASD, each including household income, parental education, insurance status, food insecurity, and neighborhood safety, along with all covariates. Results are reported as adjusted odds ratios (aORs) with 95% confidence intervals (CIs). Income and education categories were modeled ordinally to assess graded associations.

Model-based predicted probabilities were estimated to illustrate socioeconomic gradients, holding covariates at representative values. Sensitivity analyses included: (1) exclusion of children with comorbid ADHD and ASD; (2) ordered logistic regression models examining caregiver-rated severity; and (3) exploratory interaction analyses to assess potential effect modification by race/ethnicity and insurance status. Interaction findings were interpreted cautiously and presented as hypothesis-generating rather than confirmatory.

Multicollinearity was assessed using variance inflation factors. Overall model fit was evaluated using survey-adjusted Wald tests and goodness-of-fit procedures appropriate for complex survey data [11]. All analyses were conducted using statistical software capable of complex survey estimation.

### 2.6. Ethical Considerations

This study involved secondary analysis of publicly available, de-identified data. No direct interaction with human participants occurred, and no identifiable private information was accessed. In accordance with U.S. federal regulations (45 CFR §46.104(d)(4)), the study met criteria for exemption from institutional review board review.

## 3. Results

### 3.1. Sample Characteristics

The pooled analytic sample included 205,480 children aged 3–17 years, representing approximately 73.1 million U.S. children after application of survey weights. The mean age was 10.6 years (SD 4.1). Slightly more than half of the children were male (51.2%), and 48.8% were female.

By race and ethnicity, 50.8% of children were non-Hispanic White, 13.6% non-Hispanic Black, 24.1% Hispanic, and 11.5% other or multiracial. Most children lived in two-parent households (61%), followed by single-parent households (33%). Approximately 19.7% of children lived in households below 100% of the federal poverty level (FPL), while 23.1% lived in households at or above 400% FPL. Food insecurity was reported in 10.8% of households, and 4.1% of caregivers reported that their neighborhood was not safe for children to play outside.

Table 1 presents weighted demographic, socioeconomic, and neighborhood characteristics of the study population.

### 3.2. Prevalence of ADHD, ASD, and Comorbidity

The weighted prevalence of current ADHD was 9.7% (95% CI: 9.3–10.1), and the prevalence of current ASD was 2.9% (95% CI: 2.7–3.2). An estimated 1.1% of children had comorbid ADHD and ASD.

Prevalence differed markedly by sex. ADHD was reported for 13.3% of boys compared with 6.0% of girls, while ASD prevalence was 4.1% among boys and 1.1% among girls (*p* < 0.001 for both).

Transparent socioeconomic gradients were observed. ADHD prevalence declined steadily from 13.4% among children in households below 100% FPL to 6.2% among those at ≥400% FPL. ASD prevalence showed a similar pattern, decreasing from 3.8% to 2.0% across the same income range. Higher prevalence of both conditions was also observed among children whose parents had lower educational attainment, those with public insurance, those living in food-insecure households, and those residing in neighborhoods perceived as unsafe.

Children with comorbid ADHD and ASD consistently exhibited the highest prevalence within disadvantaged social groups, particularly among households with low income, food insecurity, and unsafe neighborhood conditions.

Weighted prevalence estimates across social determinants are summarized in Table 2.

### 3.3. Adjusted Associations with ADHD

In survey-weighted logistic regression models adjusting for age, sex, race/ethnicity, region, family structure, survey year, and all social determinants, multiple indicators of socioeconomic disadvantage remained associated with ADHD.

Compared with children living in households at ≥400% FPL, children in families below 100% FPL had more than twice the odds of ADHD (aOR 2.11; 95% CI: 1.82–2.46). A graded income pattern was observed across intermediate income categories. Lower parental education was also associated with higher odds of ADHD, with children whose parents had less than a high school education showing substantially higher adjusted odds than those whose parents held a bachelor’s degree or higher.

Public insurance coverage, household food insecurity, and residence in neighborhoods rated as not safe were each independently associated with higher odds of ADHD. Boys had significantly higher odds of ADHD than girls. After adjustment, non-Hispanic Black children had slightly higher odds of ADHD than non-Hispanic White children, while Hispanic children had lower adjusted odds.

### 3.4. Adjusted Associations with ASD

Adjusted models for ASD revealed similar but not identical patterns. Children living in households below 100% FPL had higher odds of ASD than those at ≥400% FPL (aOR 1.73; 95% CI: 1.32–2.28). Lower parental education, food insecurity, and unsafe neighborhood conditions were also associated with higher odds of ASD.

Sex differences were pronounced, with boys having nearly four times the odds of ASD compared with girls, and in contrast to ADHD, non-Hispanic Black and Hispanic children had substantially lower adjusted odds of ASD than non-Hispanic White children. These findings persisted after adjustment for socioeconomic position and insurance type.

Adjusted odds ratios for ADHD and ASD are presented in Table 3.

### 3.5. Predicted Probabilities and Figures

Model-based predicted probabilities illustrated strong social gradients. The estimated probability of ADHD declined from approximately 12.9% among children living below 100% FPL to 6.5% among children at ≥400% FPL. Predicted probabilities for ASD declined from 3.6% to 2.1% across the same income gradient.

Forest plots of adjusted odds ratios by race and ethnicity highlighted contrasting diagnostic patterns for ADHD and ASD. While ADHD odds were similar or slightly higher among some minoritized groups, ASD odds were consistently lower among non-Hispanic Black and Hispanic children relative to non-Hispanic White children.

**Figure 1:**
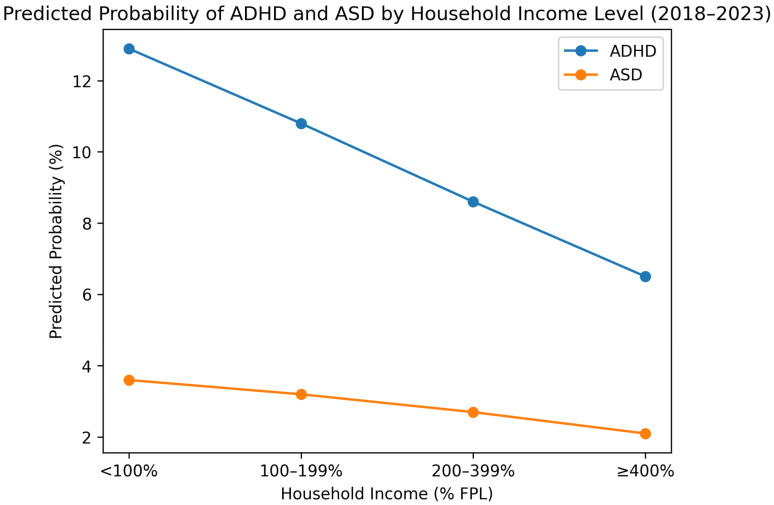
Predicted probability of ADHD and ASD by household income level.

**Figure 2:**
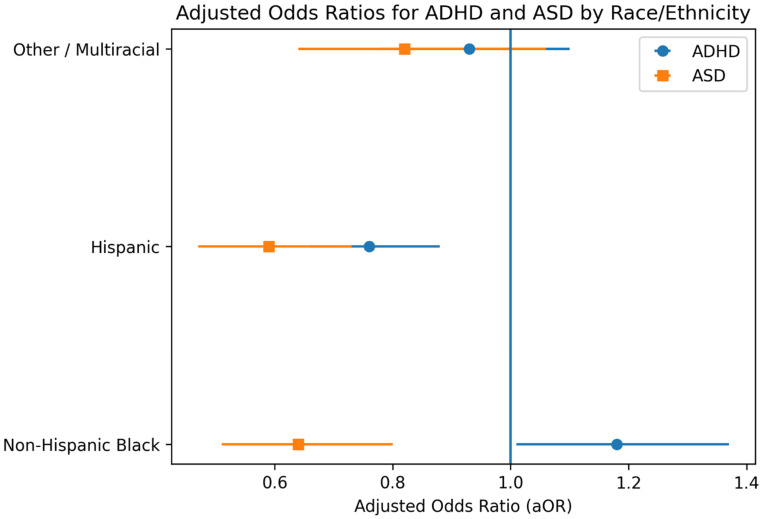
Adjusted odds ratios (95% CI) for ADHD and ASD by race/ethnicity.

These patterns are shown in Figure 1 and Figure 2.

### 3.6. Sensitivity and Exploratory Analyses

Sensitivity analyses excluding children with comorbid ADHD and ASD yielded results similar in direction and magnitude to the primary models, indicating that observed associations were not driven solely by comorbidity.

Ordered logistic regression models examining caregiver-rated severity showed that lower household income and food insecurity were associated with higher odds of moderate or severe ADHD or ASD compared with mild presentations.

Exploratory interaction analyses suggested that income gradients were steeper among non-Hispanic White children than among some other racial and ethnic groups, and that associations between neighborhood safety and ADHD were stronger among publicly insured children. These findings are interpreted cautiously and are presented as hypothesis-generating.

## 4. Discussion

### 4.1. Summary of Principal Findings

Using six consecutive cycles (2018–2023) of nationally representative data, this study examined how multiple social determinants of health are associated with attention-deficit/hyperactivity disorder (ADHD), autism spectrum disorder (ASD), and their comorbidity among U.S. children aged 3–17 years. Several consistent patterns emerged. First, both ADHD and ASD demonstrated clear socioeconomic gradients, with higher prevalence and higher adjusted odds among children living in lower-income households, experiencing food insecurity, and residing in neighborhoods perceived as unsafe. Second, these associations persisted after adjustment for demographic characteristics, family structure, geographic region, and survey year, indicating that they reflect broad population-level patterning rather than isolated subgroup effects. Third, children with comorbid ADHD and ASD were disproportionately concentrated in socially disadvantaged contexts, suggesting cumulative developmental vulnerability. Finally, pronounced sex differences and contrasting racial and ethnic patterns were observed, particularly for ASD.

Together, these findings reinforce the view that ADHD and ASD are not randomly distributed across the child population but are socially patterned conditions whose identification and lived impact are closely intertwined with family and neighborhood environments.

### 4.2. Socioeconomic and Neighborhood Mechanisms

The observed associations between socioeconomic disadvantage and neurodevelopmental disorders are consistent with a substantial body of developmental and public health research. Economic hardship can shape children’s developmental environments through multiple pathways, including increased household stress, reduced access to enriching activities, constrained caregiving resources, and exposure to adverse experiences [1,2,3]. Chronic stress associated with poverty and material hardship has been linked to alterations in attention regulation, emotional processing, and behavioral control, which may exacerbate functional difficulties or heighten caregiver concern [4].

Neighborhood context represents an important and often underappreciated dimension of these pathways. Caregiver-reported neighborhood safety likely reflects exposure to violence, disorder, or social disorganization, conditions that can restrict children’s opportunities for outdoor play, peer interaction, and autonomous exploration [5]. Such environmental constraints may interact with individual vulnerabilities to influence both behavioral expression and parental help-seeking, thereby shaping pathways to diagnosis. The strong and independent associations observed with food insecurity further underscore the role of material hardship as a marker of broader household instability rather than as an isolated nutritional risk [2,6].

Importantly, these findings should not be interpreted as evidence that social disadvantage causes ADHD or ASD. Instead, they highlight how social conditions may shape developmental experiences, functional impairment, and access to identification and services, contributing to observed population gradients.

### 4.3. Racial, Ethnic, and Diagnostic Equity Considerations

Racial and ethnic patterns observed in this study warrant careful interpretation. After adjustment for socioeconomic position and neighborhood context, non-Hispanic Black and Hispanic children had substantially lower odds of ASD compared with non-Hispanic White children. This pattern is consistent with prior research demonstrating delayed diagnosis, underdiagnosis, and reduced access to specialty services among racial and ethnic minority children [7,8,9]. Differences in developmental screening practices, referral pathways, school resources, and culturally responsive care have been widely cited as contributing factors [10,11].

For ADHD, racial and ethnic differences were less pronounced and more heterogeneous, reflecting the complex interplay of behavioral norms, school expectations, referral practices, and structural bias within educational and health systems [12]. Taken together, these findings support an interpretation centered on differential identification and access to diagnostic services rather than differences in underlying neurodevelopmental risk.

From an equity perspective, these patterns highlight the importance of culturally responsive screening and evaluation practices, as well as the need to address structural barriers that limit timely identification and support for children from historically marginalized communities.

### 4.4. Comorbidity and Cumulative Vulnerability

The disproportionate concentration of comorbid ADHD and ASD among children experiencing multiple forms of social disadvantage is a significant finding. Comorbidity has been associated with greater functional impairment, more complex clinical presentations, and higher service needs than either condition alone [13]. The observed social patterning of comorbidity suggests that overlapping diagnoses may reflect cumulative developmental risk shaped by both individual vulnerabilities and contextual stressors.

Despite its clinical significance, comorbidity is often underexamined in population-based studies. By explicitly modeling comorbid ADHD and ASD, this study contributes evidence that children with overlapping diagnoses may represent a subgroup with heightened social and developmental needs. Failure to consider comorbidity alongside social context may underestimate the level of coordinated and sustained support required by some families.

### 4.5. Implications for Policy and Practice

These findings have several implications for policy and practice. First, they support integrating developmental and behavioral screening with systematic assessment of social risks in pediatric, early childhood, and school-based settings. Identifying children who experience both developmental challenges and socioeconomic hardship may allow for earlier, more targeted interventions.

Second, the strong associations with neighborhood safety and food insecurity underscore the need for cross-sector approaches that extend beyond clinical care. Policies that promote family economic stability, expand access to food assistance, and improve neighborhood conditions may have downstream benefits for child development and well-being [1,6]. Health systems and schools can play a role by strengthening referral pathways to social services and community resources.

Finally, efforts to reduce diagnostic inequities—such as culturally responsive screening tools, provider training to address implicit bias, and improved access to specialty services—are essential for ensuring that children from all backgrounds have equitable opportunities for evaluation and support.

### 4.6. Limitations

Several limitations should be acknowledged. First, the cross-sectional design precludes conclusions about causality or temporal ordering. It is possible that caring for a child with ADHD or ASD influences parental employment, income, and stress, leading to bidirectional relationships between social conditions and diagnosis. Second, ADHD and ASD status and severity were based on caregiver reports of clinician diagnoses, which may be influenced by recall, access to evaluation, and cultural perceptions of child behavior.

Third, some social determinants, particularly neighborhood safety, rely on subjective caregiver perceptions. While these measures capture meaningful aspects of lived experience, they may vary across cultural or social contexts. Fourth, the NSCH does not capture certain potentially relevant factors, such as prenatal exposures, parental mental health, environmental toxins, or detailed service histories, which may confound observed associations. Finally, pooling pre-pandemic and intra-pandemic survey years may introduce residual heterogeneity, despite adjustment for survey year.

## 5. Conclusions

In this nationally representative analysis of U.S. children, ADHD and ASD were systematically patterned by family socioeconomic conditions and neighborhood context rather than being evenly distributed across the population. Children living in lower-income households, experiencing food insecurity, and residing in unsafe neighborhoods consistently showed higher prevalence and higher adjusted odds of both conditions. Racial and ethnic patterns, particularly for ASD, were most consistent with differential identification and access to diagnostic services rather than differences in underlying risk. The disproportionate concentration of comorbid ADHD and ASD among socially disadvantaged children highlights the importance of considering cumulative developmental and contextual vulnerability.

Overall, these findings reinforce the need to view neurodevelopmental disorders within a broader social and structural framework. Efforts to reduce disparities in ADHD and ASD will likely require coordinated strategies that combine high-quality clinical care with policies and interventions that address the social conditions shaping child development and access to services.

## Figures and Tables

**Table 1:** Weighted demographic, socioeconomic, and neighborhood characteristics of U.S. children aged 3–17 years, NSCH 2018–2023.

Characteristic	Category	Weighted %	95% CI
Sex	Male	51.2	50.4–52.0
	Female	48.8	48.0–49.6
Race/Ethnicity	Non-Hispanic White	50.8	49.6–52.0
	Non-Hispanic Black	13.6	12.9–14.3
	Hispanic	24.1	23.1–25.1
	Other/Multiracial	11.5	10.8–12.2
Household Income (% FPL)	<100% FPL	19.7	18.6–20.8
	100–199% FPL	25.4	24.2–26.6
	200–399% FPL	31.8	30.4–33.2
	≥400% FPL	23.1	22.0–24.3
Parental Education	<High school	9.5	8.8–10.2
	High school diploma	29.1	28.0–30.3
	Some college	27.6	26.4–28.8
	Bachelor’s degree or higher	33.8	32.4–35.2
Health Insurance Type	Private	54.2	53.0–55.4
	Public (Medicaid/CHIP)	38.5	37.2–39.8
	Uninsured	7.3	6.8–7.9
Neighborhood Safety	Definitely safe	70.1	69.1–71.2
	Somewhat safe	25.8	24.7–26.9
	Not safe	4.1	3.7–4.6
Food Insecurity	Yes	10.8	10.1–11.6
	No	89.2	88.4–90.0

Weighted demographic, socioeconomic, and neighborhood characteristics of U.S. children aged 3–17 years (unweighted *n* = 205,480), pooled from the 2018–2023 National Survey of Children’s Health. All estimates incorporate NSCH complex survey weights, strata, and primary sampling units. FPL = federal poverty level; CHIP = Children’s Health Insurance Program.

**Table 2:** Weighted prevalence (%) of ADHD and ASD by social determinants, U.S. children aged 3–17 years, NSCH 2018–2023.

Social Determinant	Category	ADHD % (95% CI)	ASD % (95% CI)
Household Income (% FPL)	<100% FPL	13.4 (12.1–14.7)	3.8 (3.1–4.5)
	100–199% FPL	10.8 (9.8–11.8)	3.2 (2.6–3.8)
	200–399% FPL	8.6 (7.8–9.4)	2.7 (2.3–3.1)
	≥400% FPL	6.2 (5.6–6.8)	2.0 (1.7–2.3)
Parental Education	<High school	14.6 (12.8–16.4)	3.9 (3.1–4.7)
	High school diploma	11.2 (10.1–12.3)	3.3 (2.7–3.9)
	Some college	9.0 (8.1–9.9)	2.7 (2.3–3.1)
	Bachelor’s degree or higher	6.8 (6.1–7.5)	2.1 (1.8–2.4)
Health Insurance Type	Private	8.1 (7.5–8.7)	2.4 (2.1–2.7)
	Public (Medicaid/CHIP)	13.2 (12.0–14.4)	3.8 (3.2–4.4)
	Uninsured	10.5 (8.4–12.6)	2.6 (1.8–3.4)
Neighborhood Safety	Definitely safe	8.9 (8.3–9.5)	2.5 (2.2–2.8)
	Somewhat safe	12.5 (11.3–13.7)	3.4 (2.8–4.0)
	Not safe	15.8 (13.0–18.6)	4.4 (3.0–5.8)
Food Insecurity	Yes	14.9 (13.2–16.6)	4.1 (3.4–4.9)
	No	8.8 (8.3–9.3)	2.7 (2.4–3.0)

Weighted prevalence of parent-reported, clinician-diagnosed current attention-deficit/hyperactivity disorder (ADHD) and autism spectrum disorder (ASD) across selected social determinants of health among U.S. children aged 3–17 years. Estimates are based on pooled 2018–2023 National Survey of Children’s Health data and incorporate complex survey weights, strata, and primary sampling units. FPL = federal poverty level; CHIP = Children’s Health Insurance Program.

**Table 3:** Adjusted odds ratios (aORs) for ADHD and ASD by social determinants, U.S. children aged 3–17 years, NSCH 2018–2023.

Determinant	Category	ADHD aOR (95% CI)	ASD aOR (95% CI)
Household Income (% FPL)	<100% FPL	2.11 (1.82–2.46)	1.73 (1.32–2.28)
	100–199% FPL	1.69 (1.46–1.95)	1.32 (1.04–1.67)
	200–399% FPL	1.28 (1.12–1.45)	1.14 (0.92–1.40)
	≥400% FPL (ref)	1.00	1.00
Parental Education	<High school	1.83 (1.52–2.20)	1.46 (1.12–1.91)
	High school diploma	1.42 (1.26–1.60)	1.28 (1.01–1.63)
	Some college	1.21 (1.07–1.36)	1.10 (0.88–1.37)
	Bachelor’s degree or higher (ref)	1.00	1.00
Health Insurance Type	Public (Medicaid/CHIP)	1.37 (1.20–1.55)	1.21 (0.98–1.49)
	Uninsured	1.18 (0.95–1.47)	1.05 (0.72–1.52)
	Private (ref)	1.00	1.00
Neighborhood Safety	Somewhat safe	1.31 (1.15–1.50)	1.24 (0.97–1.59)
	Not safe	1.94 (1.51–2.49)	1.79 (1.21–2.64)
	Definitely safe (ref)	1.00	1.00
Food Insecurity	Yes	1.88 (1.60–2.21)	1.61 (1.18–2.19)
	No (ref)	1.00	1.00
Child Sex	Male	2.39 (2.12–2.68)	3.84 (3.13–4.72)
	Female (ref)	1.00	1.00
Race/Ethnicity	Non-Hispanic Black	1.18 (1.01–1.37)	0.64 (0.51–0.80)
	Hispanic	0.76 (0.66–0.88)	0.59 (0.47–0.73)
	Other/Multiracial	0.93 (0.78–1.10)	0.82 (0.64–1.06)
	Non-Hispanic White (ref)	1.00	1.00

Survey-weighted adjusted odds ratios (aORs) and 95% confidence intervals (CIs) for parent-reported, clinician-diagnosed current ADHD and ASD among U.S. children aged 3–17 years. Models adjust for age, sex, race/ethnicity, family structure, U.S. Census region, and survey year, and incorporate NSCH complex survey weights, strata, and primary sampling units. FPL = federal poverty level; CHIP = Children’s Health Insurance Program; ref = reference category.

## Data Availability

National Survey of Children’s Health (NSCH) datasets are publicly available at: https://www.childhealthdata.org/dataset (accessed on 15 December 2025). Complementary surveillance datasets (NHIS, ADDM) are publicly available from the Centers for Disease Control and Prevention: https://www.cdc.gov (accessed on 15 December 2025).
